# Frequency and Focus of in Vitro Studies of Microglia-Expressed Cytokines in Response to Viral Infection: A Systematic Review

**DOI:** 10.1007/s10571-024-01454-9

**Published:** 2024-02-13

**Authors:** Diego A. Barrios-González, Santiago Philibert-Rosas, Iris E. Martínez-Juárez, Fernando Sotelo-Díaz, Verónica Rivas-Alonso, Julio Sotelo, Mario A. Sebastián-Díaz

**Affiliations:** 1 Epilepsy Clinic. National Institute of Neurology and Neurosurgery, Mexico City, Mexico; 2 Multiple Sclerosis Clinic, National Institute of Neurology and Neurosurgery, Mexico City, Mexico; 3 Department of Neuroimmunology, National Institute of Neurology and Neurosurgery, Mexico City, Mexico; 4 Nephrology Department, South Central High Specialty Hospital PEMEX, Anillo Periférico 4019 Fuentes del Pedregal, Tlalpan, 1440 Mexico City, Mexico

**Keywords:** Cytokines, Microglia, In Vitro, Viral infection

## Abstract

It is well known that as part of their response to infectious agents such as viruses, microglia transition from a quiescent state to an activated state that includes proinflammatory and anti-inflammatory phases; this behavior has been described through in vitro studies. However, recent in vivo studies on the function of microglia have questioned the two-phase paradigm; therefore, a change in the frequency of in vitro studies is expected. A systematic review was carried out to identify the microglial cytokine profile against viral infection that has been further evaluated through in vitro studies (pro-inflammatory or anti-inflammatory), along with analysis of its publication frequency over the years. For this review, 531 articles published in the English language were collected from PubMed, Web of Science, EBSCO and ResearchGate. Only 27 papers met the inclusion criteria for this systematic review. In total, 19 cytokines were evaluated in these studies, most of which are proinflammatory; the most common are IL-6, followed by TNF-α and IL-1β. It should be pointed out that half of the studies were published between 2015 and 2022 (raw data available in https://github.com/dadriba05/SystematicReview.git). In this review, we identified that evaluation of pro-inflammatory cytokines released by microglia against viral infections has been performed more frequently than that of anti-inflammatory cytokines; additionally, a higher frequency of evaluation of the response of microglia cells to viral infection through in vitro studies from 2015 and beyond was noted.

**Graphical Abstract:**

In vitro assessment of microglia-released cytokines upon viral infection has been more frequent since 2015 and has focused more on pro-inflammatory cytokines.

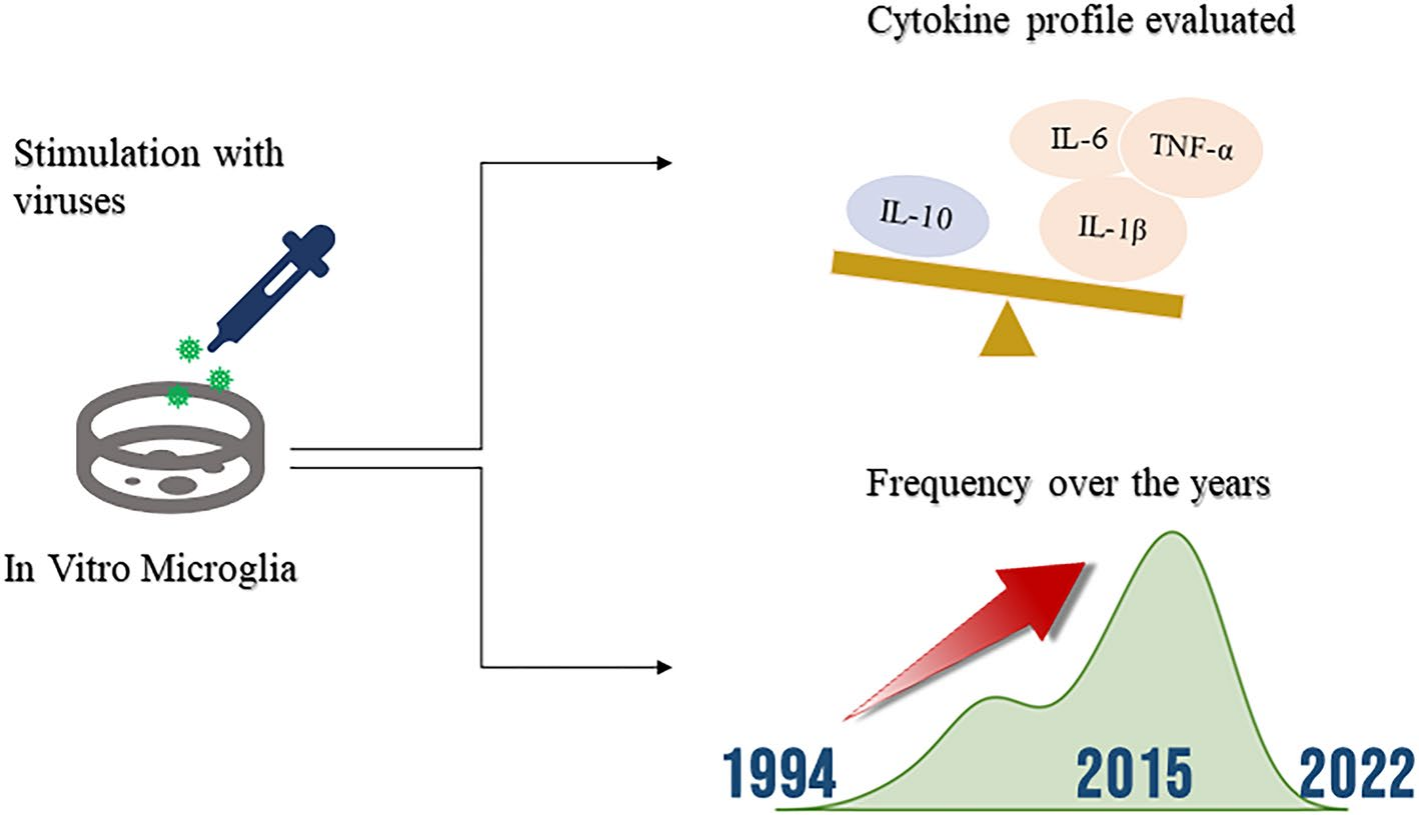

## Introduction

Microglia are cells of the central nervous system with immune function and are found in all regions of the brain. The response of these cells to invading agents such as viruses consists of transition from a quiescent state to an activated state in which they acquire amoeboid morphology and release cytokines to participate in the immune response. This cytokine release process is formed, according to in vitro studies, by a “classical” phase with release of proinflammatory cytokines and an “alternative” phase with release of anti-inflammatory cytokines (Michelucci et al. 2009; Sargsyan et al. 2011; Xiao et al. 2007). However, in vivo studies describe findings that do not coincide with this two-phase paradigm (Chiu et al. 2013; Wes et al. 2016), which could result in a change in the frequency of in vitro studies on the microglial response to infectious agents, a phenomenon thus far not reported in the literature and identification of which may be useful to improve and guide preclinical research in this domain.

On the other hand, cytokines released to generate an inflammatory response against the invader also have deleterious effects on the central nervous system (CNS), damaging the blood‒brain barrier by cytotoxic effects, particularly through proinflammatory cytokines. However, as the anti-inflammatory cytokines also released by microglia partially inhibit the pro-inflammatory state, it is relevant to study this alternating profile (Colonna and Butovsky 2017).

The systematic review in this report aims to identify studies that have evaluated the cytokines expressed by microglia in culture against a viral presence to analyze which cytokine profile is the most assessed (proinflammatory or anti-inflammatory) and how often this type of in vitro study has been reported.

## Methods

### Search Strategy and Selection Criteria

A systematic review was conducted to identify the frequency of in vitro studies evaluating cytokines expressed by cultured microglia against stimulation with viruses, as well as cytokines that are commonly assessed by such studies. The articles included are reports that assessed the response of microglial cultures to viral infection. This systematic review was conducted under the guidelines of “Cochrane Handbook for Systematic Review of Interventions” and those of “Preferred Reporting Items for Systematic Reviews and Meta-Analyses” (PRISMA).

We performed a sensitive search based on the use of keywords and groups of synonyms, which included articles in the English language and was carried out in the databases PubMed, Web of Science, EBSCO and ResearchGate by using the keywords “Microglia”, “Cytokines”, “Virus Infection/Disease”, “In Vitro” and “Cultures” to identify manuscripts that met the eligibility criteria. In the PubMed database, the resource “Mesh” was used to favor the search with the established keywords.

**Fig. 1:**
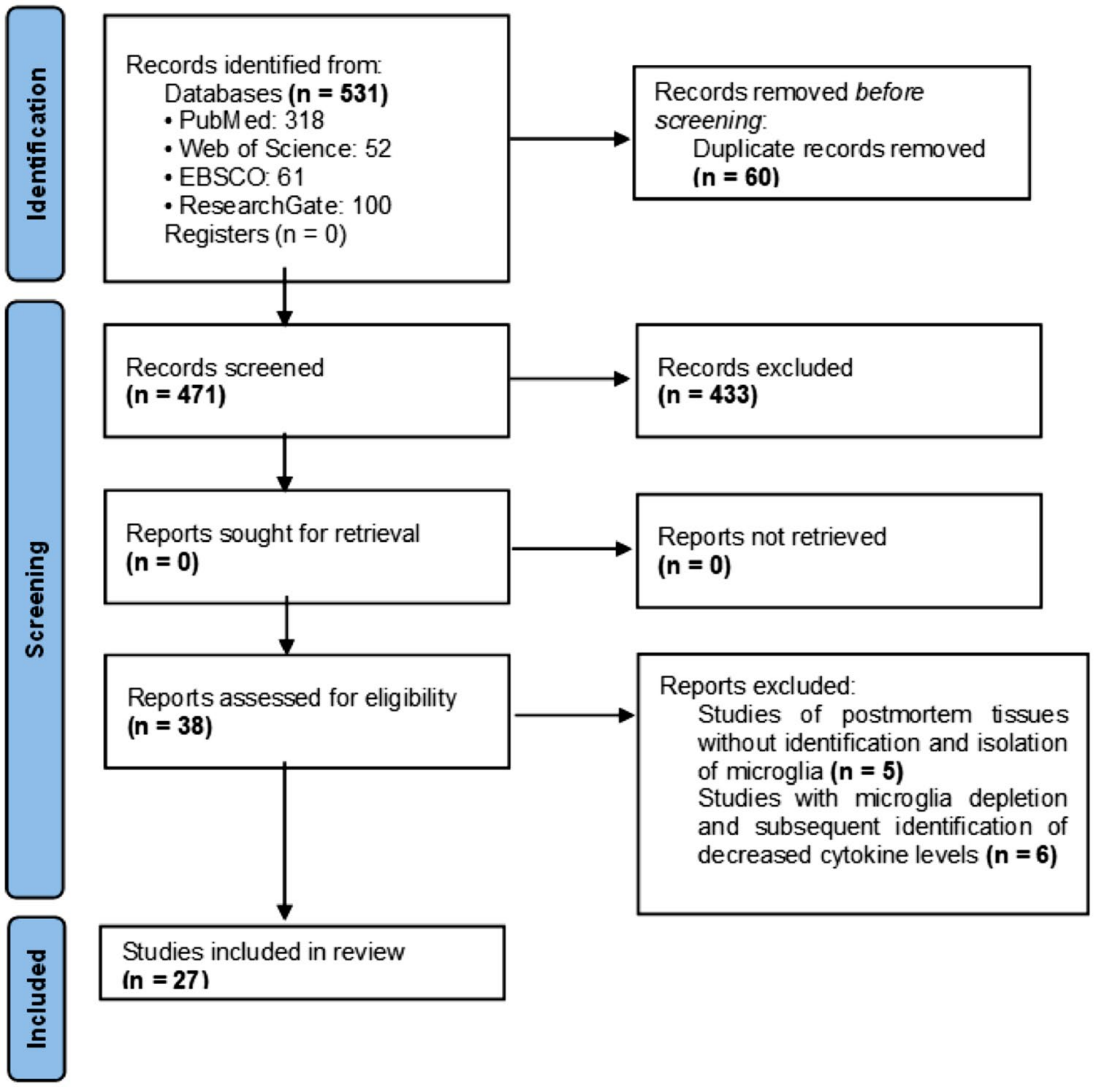
The PRISMA flowchart

The included reports describe in vitro studies of animal or human microglia directly stimulated with viruses or infected macrophages and the cytokines expressed in response, which were identified either by genetic testing of mRNA or by immunohistochemical methods. Studies of postmortem samples without microglia isolation were not included, nor were studies that evaluated the effects of microglia suppression and consequent changes in cytokine concentration (Fig. 1).

To identify expression of cytokines in response to viral stimulus, an increase in cytokine concentration with respect to baseline was considered a positive cell response. Cohort points with statistically significant values were established according to the method used to identify cytokines and were individualized for each study.

### Risk of Bias Assessment

The risk of bias of the studies included in the review was not evaluated due to the lack of a standardized tool with criteria for in vitro studies. We did not register the protocol in International Prospective Register of Systematic Reviews, but it is available upon request.

### Statistical Analysis and Data Availability

A descriptive analysis of the data was performed, for which a database was established in the statistical program IBM SPSS (version 26) ®. The cytokines expressed by microglia in each study were considered nominal variables, and the year of publication of each included report was considered a quantitative variable. Once statistical analysis was performed, the data are provided in tables and plots created with IBM SPSS (version 26) ® and JASP ®. Raw data are available at a public repository in GitHub®: https://github.com/dadriba05/SystematicReview.git.

## Results

### Literature Search

A total of 531 reports were found, of which 60 were removed prior to screening for duplication. We excluded 433 studies because their focus was to evaluate a change in the morphology of microglia rather than expression of cytokines by stimulation with viruses. In total, 38 studies were eligible, of which 11 were further excluded: 5 due to cytokine assessment in postmortem samples without isolating microglia and 6 for analyzing studies already included in the review (Fig. 1). The characteristics of the included studies are shown in Table 1.

**Table 1:** Characteristics of the studies included in the systematic review. RT‒PCR: Real Time-PCR; IHC: Immunohistochemistry

Year of publication	Sample type	Cytokine identification method	Virus	References
1994	Human microglia	RT‒PCR	MV	(Yamabe et al. 1994)
2000	Sheep microglia	RT‒PCR	MVV	(Ebrahimi et al. 2000)
2001	Human microglia	Direct IHC	CMV	(M. C.-J. Cheeran et al. 2001)
2001	Human microglia	Direct IHC	(HSV) 1	(Lokensgard et al. 2001)
2001	Mouse microglia	RT‒PCR	TMEV	(Olson et al. 2001)
2004	Mouse microglia	RT‒PCR	MHV-A59	(Li et al. 2004)
2005	Human microglia	Direct IHC	WNV	(M. C. J. Cheeran et al. 2005)
2010	Mouse microglia	Direct IHC	JEV	(C. J. Chen et al. 2010)
2011	Poultry microglia	RT‒PCR	GaHV-2	(Yang et al. 2011)
2012	Mouse microglia	Direct IHC	SINV	(Esen et al. 2012)
2012	Macaque microglia	Direct IHC	VIS	(Renner et al. 2012)
2013	Mouse microglia	RT‒PCR	RABV	(Zhao et al. 2013)
2015	Mouse microglia	RT‒PCR	DENV	(Bhatt et al. 2015)
2015	Mouse microglia	Direct IHC	EV71	(Chang et al. 2015)
2017	Mouse microglia	RT‒PCR	JEV	(Deng et al. 2017)
2017	Human microglia	Direct IHC	JEV	(Lannes et al. 2017)
2017	Human microglia	Direct IHC	ZIKV	(Lum et al. 2017)
2018	Human microglia	RT‒PCR	ZIKV	(Diop et al. 2018)
2018	Human microglia	Direct IHC	VEEV	(Keck et al. 2018)
2018	Mouse microglia	Direct IHC	ZIKV	(Wang et al. 2018)
2019	Human microglia	Direct IHC	HHV-6A	(Bortolotti et al. 2019)
2020	Mouse microglia	RT‒PCR	JEV	(Kumar et al. 2020)
2020	Human microglia	RT‒PCR	MHV-A59	(Lavi & Cong 2020)
2020	Human microglia	Direct IHC	RSV	(Zhang et al. 2021)
2021	Human microglia	RT‒PCR	SARS-CoV-2	(Mishra & Banerjea 2021)
2022	Human microglia	Direct IHC	VI A(H1N1)	(Ding et al. 2022)
2022	Human microglia	RT‒PCR	SARS-CoV-2	(Jeong et al. 2022)

### Viruses Used for in vitro Stimulation

**Fig. 2:**
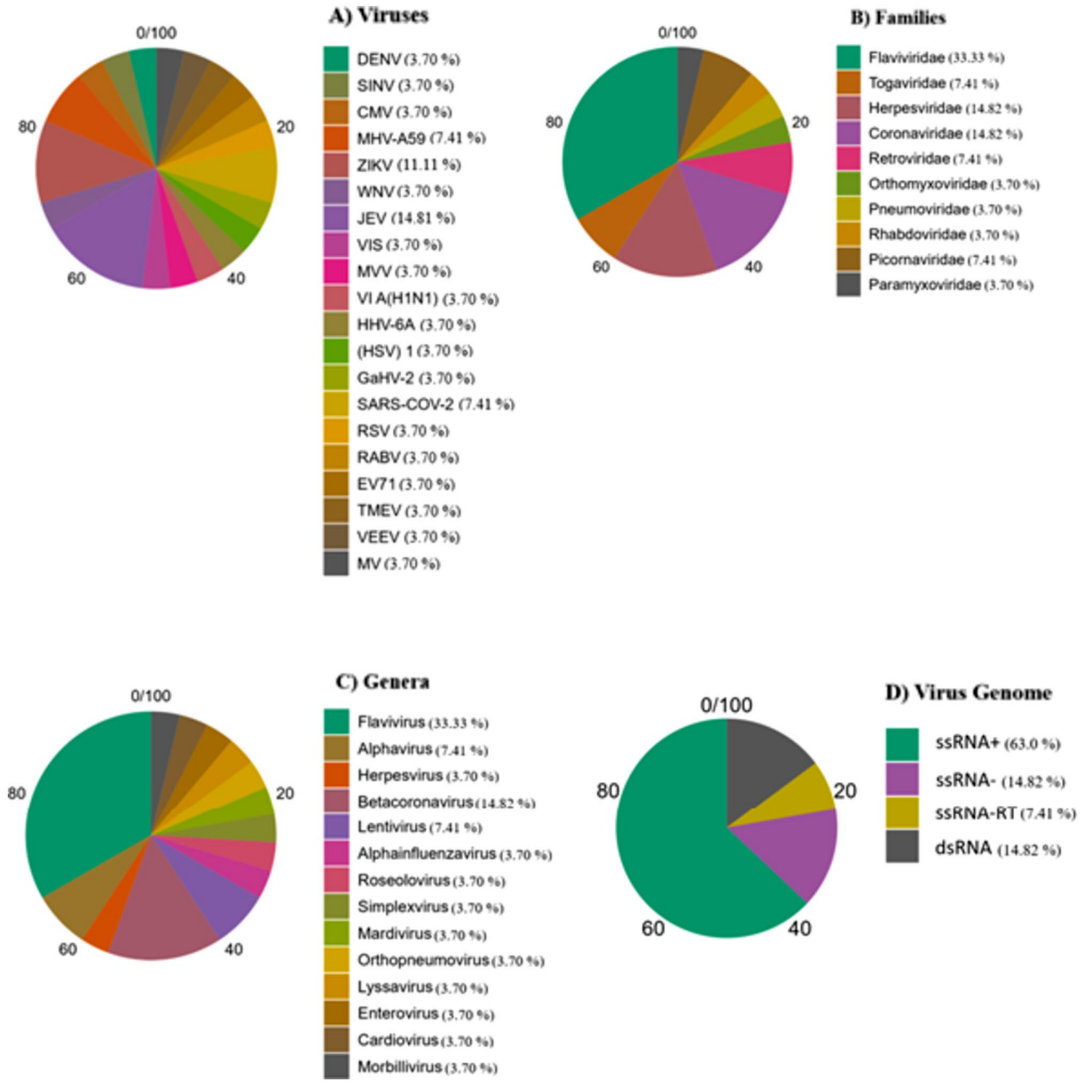
Characteristics of viruses assessed in the studies included. Percentages by **A** viruses, **B** families, **C** genera and **D** viral genome

Viral stimulation of microglia in vitro was performed with Japanese encephalitis virus (JEV) in 4 studies (14.8%), Zika virus in 3 studies (11.1%), SARS-CoV-2 in 2 studies (7.4%) and another mouse hepatitis virus strain A59 in 2 studies (MHV-A69, 7.4%). The remaining studies (3.7% for each) used a different virus. By viral family, the most used for cell stimulation was *Flaviviridae* in 9 (33.3%), followed by *Coronaviridae* in 4 (14.8%) and *Herpesviridae* in 4 (14.8%). By genus, the most frequently assessed was *Flavivirus* in 9 (33.3%) and *Betacoronavirus* in 4 (14.8%). By viral genome, the viruses assessed were predominantly ssRNA + (Fig. 2).

### Cytokines Assessed in the in vitro Studies

**Fig. 3:**
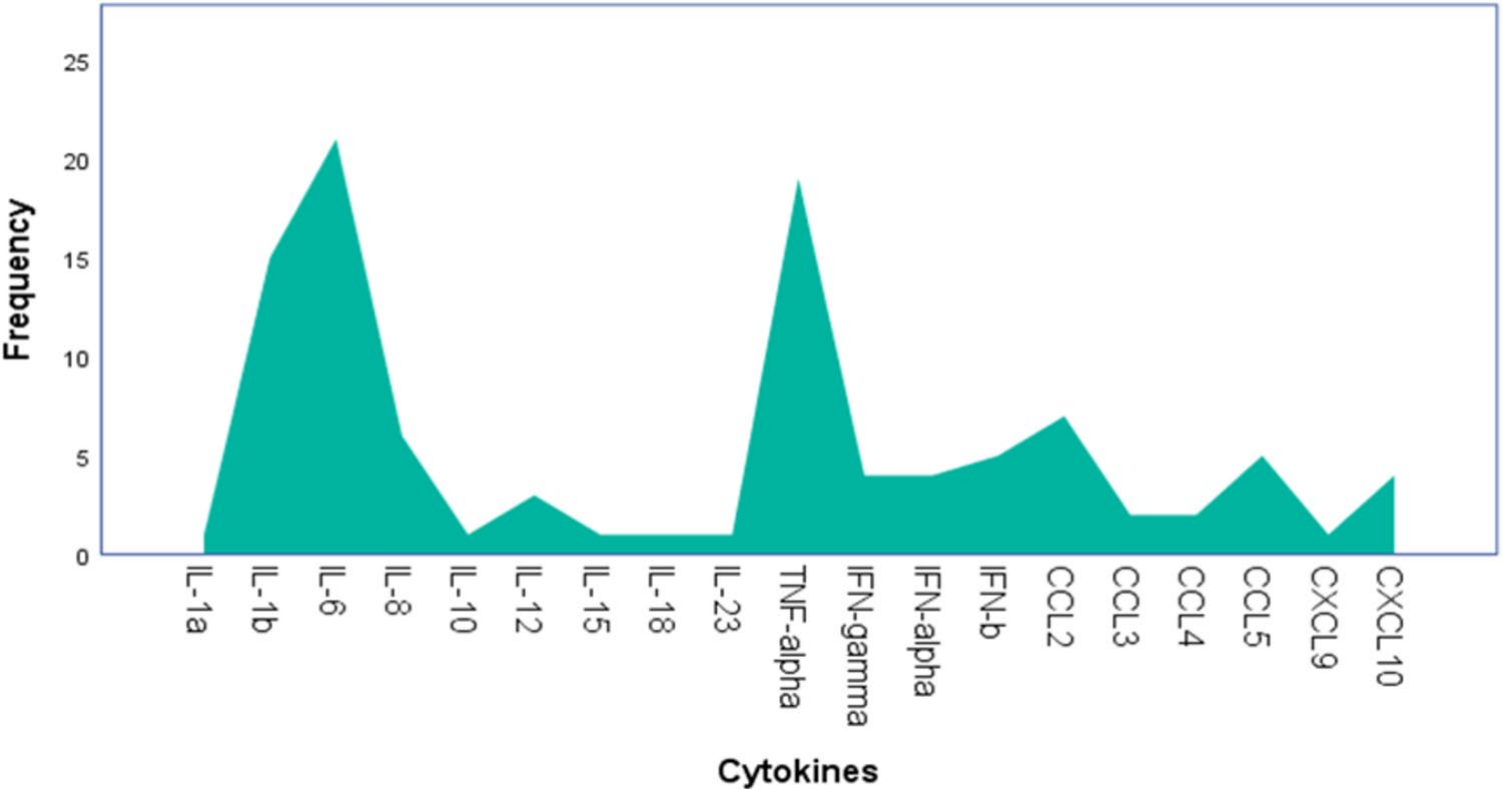
Count of the cytokines evaluated in the studies and expressed by in vitro microglia in response to viral stimulation. In total, 19 types of cytokines were evaluated and expressed by microglia. Of these, IL-10 is the only anti-inflammatory cytokine. IL-6 was the most frequently reported cytokine (20.38%), followed by TNF-α (18.44%), both with a much higher frequency than the other reported cytokines

Among the 27 studies included, 19 types of cytokines were assessed and released by microglia. IL-6 was the most frequent (20.38%), followed by TNF-α (18.44%) and IL-1β (14.56%), with these three cytokines far exceeding the others evaluated. Only one cytokine assessed (IL-10) corresponded to the anti-inflammatory profile (Fig. 3).

### Publication Frequency of in vitro Reports

**Fig. 4:**
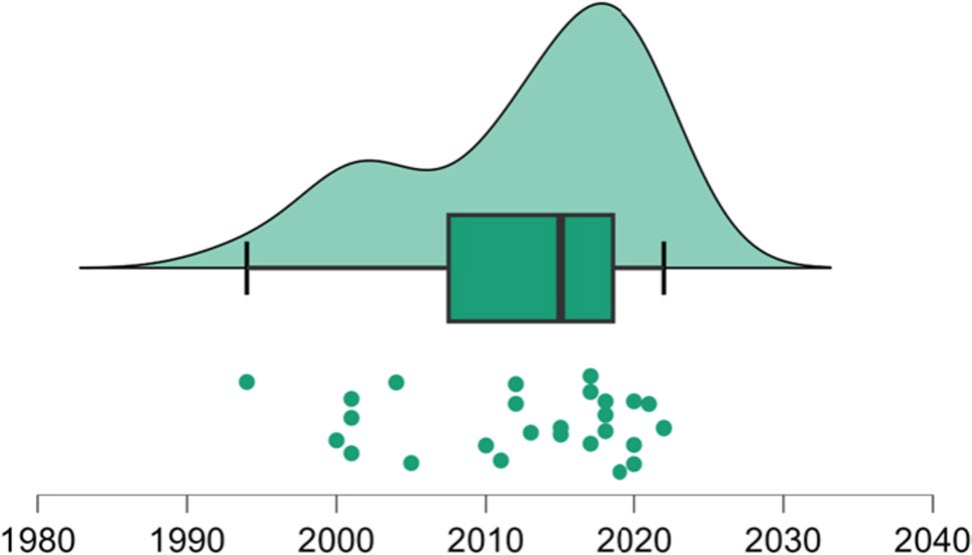
Descriptive analysis of studies’ publication year. Raincloud plot: dots represent the year in which each paper was published; the box plot shows the minimum of the data series that corresponds to year 1994, the maximum to 2022 and median (black line) to 2015 (Color figure online)

We identified the publication frequency over the years. In this distribution, the smallest number corresponds to 1994, the date of the oldest article, and the largest number corresponds to 2022, the date of publication of the most recent report. Thus, 50% of the studies were published between 1994 (minimum) and 2015 (median), and the remaining 50% were published between 2015 and 2022 (maximum) (Fig. 4).

## Discussion

This systematic review identified that in vitro evaluation of proinflammatory cytokines released by microglia against viral infections, mainly the cytokines IL-6, TNF-α and IL-1β, was frequently compared to the evaluation of anti-inflammatory cytokines. The reason for this might be due to the conceptualization of microglial function in vitro, with an activated state categorized as a first stage called “classical” or “M1”, in which microglia release proinflammatory cytokines with neurotoxic effects, followed by a second stage called “alternative” or “”M2”, with release of anti-inflammatory cytokines and healing activity (Michelucci et al. 2009; Sargsyan et al. 2011; Xiao et al. 2007). However, it is increasingly accepted that microglial function rarely has a tendency toward either of these two stages described by in vitro studies, as based on in vivo models of neurodegeneration that have identified concurrent expression of both neurotoxic and neuroprotective factors by microglia (Chiu et al. 2013; Wes et al. 2016).

Given this fact, it would be expected that studies in vitro have been replaced by those in vivo to better characterize the function of microglia. However, the observed asymmetry in the frequency distribution of the studies’ publication years shows the opposite. This distribution reveals that 50% of the studies were published between 1994 and 2015 and that the remaining percentage was published between 2015 and 2022 (Fig. 4). Therefore, starting in 2015, half of the studies were published within a shorter period, indicating the permanence of a significant frequency of research that evaluates the response of microglia to viral infection through in vitro studies.

This trend has implications for adequate characterization of the pathogenesis of viral encephalitis, as some of its complications are due to the neurotoxic effects of the immune response and the lack of an adequate compensatory response to inhibit it (Z. Chen et al. 2019). This is evident in the case of cognitive dysfunction and memory impairment, which are seen in long-term clinical studies in patients with viral encephalitis (Sadek et al. 2010; Sejvar et al. 2003), both associated with presynaptic membrane damage in the hippocampus, as mediated by microglia through complement activation (Vasek et al. 2016). Consequently, immunosuppressive therapy has emerged as a potential therapeutic alternative to antiviral treatment (Aksamit 2021), and its effectiveness is currently being evaluated in multicenter trials (Stahl 2018). Therefore, the results of in vivo studies do provide valuable information and likely more accurate information than isolated in vitro evaluations.

## Conclusion

The findings obtained in this systematic review allowed us to identify a high frequency of in vitro studies evaluating expression of microglial cytokines in response to viral stimulation, particularly since 2015. Furthermore, the majority of these studies have focused on examining pro-inflammatory cytokines, with only one report evaluating their contrasting anti-inflammatory cytokines. The context is noteworthy considering the concurrent presence of both profiles by recent in vivo studies.

This systematic review focuses on recent in vitro evaluations of cultured microglial cell responses to viral infection. By providing this characterization, the review facilitates the potential clinical application of these results in immunotherapeutic trials, contributing to the improvement of the safety and efficacy of therapeutic alternatives for disorders due to viral infection of the brain. Additionally, we show the lack of a standardized tool for assessing the risk of bias in in vitro studies. This gap in the evaluation of preclinical evidence can lead to inappropriate applications of results to clinical practice.

Based on the aforementioned, it is crucial to promote in vivo studies for a more comprehensive characterization of the microglial response to viral infection. This is necessary to address existing gaps regarding the theory of distinct phases (classical and alternative) of the microglial response to viral infection and to better differentiate the anti-inflammatory response in viral encephalitis. The development of a standardized tool for assessing in vitro studies is essential. Such a tool would enhance critical methodological assessment of these studies and have a significant impact on the clinical application of experimental studies.

## Data Availability

Raw data are available on a public repository in GitHub®: https://github.com/dadriba05/SystematicReview.git.
